# Cherokee County Medical Society

**Published:** 1890-12

**Authors:** J. K. Frazer


### CHEROKEE COUNTY MEDICAL SOCIETY.

*Editor Daniel's Texas Medical Journal:*

The Cherokee County Medical Society met in regular session at Jacksonville last month. Owing to the ill health of Dr. McCord the committee on collective investigation made no report. Three regular papers, however, were read. One by Dr. Evans,

†Published in the November number of the Journal.—Ed.

on Sterility; by Dr. Guinn, of Rusk, on Gonorrhœa, and one on Drunkenness, by Dr. Cavanness.

These papers were well timed and freely discussed, and discussions are all voluntary, free and easy. This meeting was very well attended and was one (like all the rest) of interest and profit. Our Association has accomplished, and is still accomplishing good in our county. We took in two new members, leaving only nine in the county on the *outside*.

The subjects and essayists for next meeting are: "Hyperplasia of the Uterus," by Dr. F. E. Fuller. "Causes, Diagnosis and Treatment of Endometritis," by Dr. A. H. McCord. "Causes and Treatment of Cervix Uteri," [?] by Dr. J.W. Smith. "Causes and Treatment of Acute Yellow Atrophy of the Liver," by Dr. J. F. Johnson.

Our next meeting is in January, at which time we hope to have a full attendance.

Yours truly,

J. K. FRAZER, M. D.

